# How does plasticity of migration help tumor cells to avoid treatment: Cytoskeletal regulators and potential markers

**DOI:** 10.3389/fphar.2022.962652

**Published:** 2022-10-06

**Authors:** Antonina Alexandrova, Maria Lomakina

**Affiliations:** N.N. Blokhin National Medical Research Center of Oncology, Institute of Carcinogenesis, Moscow, Russia

**Keywords:** actin cytoskeleton, metastasis, EMT, actin-binding proteins, motility modes, hypoxia

## Abstract

Tumor shrinkage as a result of antitumor therapy is not the only and sufficient indicator of treatment success. Cancer progression leads to dissemination of tumor cells and formation of metastases - secondary tumor lesions in distant organs. Metastasis is associated with acquisition of mobile phenotype by tumor cells as a result of epithelial-to-mesenchymal transition and further cell migration based on cytoskeleton reorganization. The main mechanisms of individual cell migration are either mesenchymal, which depends on the activity of small GTPase Rac, actin polymerization, formation of adhesions with extracellular matrix and activity of proteolytic enzymes or amoeboid, which is based on the increase in intracellular pressure caused by the enhancement of actin cortex contractility regulated by Rho-ROCK-MLCKII pathway, and does not depend on the formation of adhesive structures with the matrix, nor on the activity of proteases. The ability of tumor cells to switch from one motility mode to another depending on cell context and environmental conditions, termed migratory plasticity, contributes to the efficiency of dissemination and often allows the cells to avoid the applied treatment. The search for new therapeutic targets among cytoskeletal proteins offers an opportunity to directly influence cell migration. For successful treatment it is important to assess the likelihood of migratory plasticity in a particular tumor. Therefore, the search for specific markers that can indicate a high probability of migratory plasticity is very important.

## 1 Introduction

The development of targeted therapy and immuno-therapeutic strategies led to significant progress in anti-cancer treatment. At the same time, most antitumor drugs are aimed to stop the proliferation of tumor cells and/or stimulate their death. However, a shrinking of the tumor volume is not always evidence of the successful treatment, because the metastasis which led to the development of secondary tumor foci in distant organs appears to be the main cause of death in cancer patients (Sleeman and Steeg, 2010; Welch and Hurst, 2019).

The first step leading to metastasis development is turn of immobile epithelial cells to the so-called migratory phenotype (Hanahan and Weinberg, 2011), which allows these cells to detach from the primary tumor and to migrate long distances. The acquisition of migratory phenotype by tumor cells in solid tumors is achieved as a result of epithelial-to-mesenchymal transition (EMT) (Polyak and Weinberg, 2009), which is considered one of the hallmarks of tumor progression (Hanahan and Weinberg, 2011).

Disseminating cancer cells (CC) can migrate from the primary tumor in clusters or as individual cells, using either mesenchymal or amoeboid mode of migration (Friedl and Wolf, 2010; Pandya et al., 2017), which are regulated by different molecular mechanisms. The ability to switch from one motility mode to another depending on the cellular context and the microenvironment is named migratory plasticity. It is a feature of many CCs, allowing them to adapt to the variable environment and often escape the drug treatment (Parri et al., 2009; Friedl and Wolf, 2010).

Since the excellent reviews describing the cellular mechanisms of different motility modes and the basic transitions between them exist (Friedl and Wolf, 2003; Friedl and Wolf, 2010; Panková et al., 2010; Clark and Vignjevic, 2015; Te Boekhorst and Friedl, 2016; Paul et al., 2017; Yamada and Sixt, 2019), we will not dwell on this, but only notice very briefly the main characteristics of the various types of migration known to date, with a special emphasis on the factors that stimulate migratory plasticity. It is necessary to pay attention on these factors because underestimation of them leads to arising of side effects in the use of both new and already known drugs, which can lead to activation of migratory plasticity, increase metastasis, and thus aggravate the development of the disease. We will review the current data demonstrating the involvement of various cytoskeletal proteins in the regulation of migratory plasticity and try to analyze which side effects provoking migratory plasticity may occur when using different therapeutic approaches. The development of specific agents aimed at stopping the migration of tumor cells seems to be a very actual task. However, the solution should take into account not only the inhibition of a particular migration mode but also the possibility of mutual transitions.

Another important task is to search for markers indicating that the cells of that particular tumor could possess the plasticity of migration. This will help to consider the risk of disease complications and to choose the scheme of treatment for specific tumors to minimize such risk. In our review we will analyze recent data addressing these issues.

## 2 The modes of tumor cell dissemination and migratory plasticity

In the process of metastasis formation after undergoing EMT and detachment from a primary tumor, cells use different modes of migration, which are based on different cellular mechanisms (Friedl and Wolf, 2010; Yamada and Sixt, 2019).

A distinction is made between collective migration, in which cells keep cell-cell contacts and move in clusters, and individual migration, in which cells completely lose contacts with each other and move as single cells. Collective migration is based on the so-called hybrid EMT in which cells retain both epithelial characteristics - incomplete disappearance of E-cadherin and retention of cell-cell contacts - and acquire mesenchymal characteristics such as vimentin expression, loss of apical lateral polarization and acquire polarization in the direction of movement. It has been shown that cells with features of hybrid EMT are more prone to metastasis than cells with a fully epithelial or mesenchymal phenotype (Rubtsova et al., 2015; Pastushenko et al., 2018; Rubtsova et al., 2021).

Two main modes of single cell migration are distinguished based on the mechanisms underlying the movement: mesenchymal, which is characterized by formation of such protrusions as lamellipodia, filopodia or invadopodia upon of small GTPase Rac activation, formation of focal adhesions with the extracellular matrix (ECM) and proteolytic degradation of ECM to form migration routes (Pandya et al., 2017); and amoeboid mode, which is characterized by bleb-like protrusions formed due to Rho-dependent actin-myosin contractility, independence of cell-ECM adhesions formation and protease activity (Paluch et al., 2016). There are several description of slightly different types of amoeboid motility in recent publications: fast amoeboid motility for transformed cells and leucocytes (Liu, et al., 2015), leader bleb-based motility of melanoma cells (Logue et al., 2015), and stable bleb-based motility of early progenitor zebrafish cells (Ruprecht et al., 2015). The physical basis for all these types of migration is the same - it occurs in confinement low-adhesive conditions and requires the high actin cortex contractility and rearward cortical flow, which in combination with unspecific friction drives fast cell migration. For a detailed acquaintance with the mechanisms of amoeboid and mesenchymal movement we recommend the reviews (Friedl and Alexander, 2011; Pandya et al., 2017; Wu et al., 2021; Graziani et al., 2022). Collective migration was thought to be based mainly on mesenchymal motility mode, but recent work has shown that cells could also migrate by groups using the special type of amoeboid mechanisms (Pagès et al., 2020). One of the reasons for the development of effective metastasis as a result of tumor progression or as a response to therapy is the ability of CCs to switch between mesenchymal and amoeboid modes of migration. Since there are almost no data on transitions between mesenchymal and amoeboid modes for collectively migrated cells, we will discuss migratory plasticity in terms of exchange of migration machineries in process of single cells dissemination.

The specific mechanisms of CCs migration depend on the type, origin, and stage of the tumor. The mesenchymal migration mode was described for cells from sarcomas, gliomas, and epithelial cancers after they underwent EMT. It also was shown to be characteristics of cells derived from tumors with low levels of cell differentiation (spindle-cell tumors) (Cates et al., 2008). The amoeboid migration was shown for cells derived from myeloid leukemia, lymphoma, melanomas and some epithelial tumors, such as breast cancer, lung (small-cell lung carcinoma) and prostate cancers (Condeelis and Segall, 2003; Pinner and Sahai, 2008; Madsen and Sahai, 2010). It was shown by intravital imaging that melanoma and breast cancer cells migrate by blebbing in live mice (Tozluoglu et al., 2013).

Mesenchymal movement and the process of EMT are very well studied. Amoeboid movement and transition to amoeboid movement are somewhat less studied, although the number of researches studying the mechanisms of amoeboid movement and the triggers that provoke the mesenchymal-to-amoeboid transition (MAT) is constantly growing (Friedl and Alexander, 2011; Friedl et al., 2012; Pandya et al., 2017; Wu et al., 2021). The very different phenomenon could trigger the switches between motility modes, the summary of the latest data can be found in tables in recent reviews (Panková et al., 2010; Te Boekhorst and Friedl, 2016; Alexandrova et al., 2020). Among the triggers of MAT there are numerous factors that limit mesenchymal motility. For example, inhibition of the activity of matrix metalloproteases (MMPs), prevention of either actin based protrusions or formation of cell-substrate adhesions have been found to induce MAT. That switch allows tumor cells to continue migration in conditions unfavorable for mesenchymal motility (for reviews, see Agarwal and Zaidel-Bar, 2019; DeSimone and Horwitz, 2014; Friedl and Wolf, 2010; Friedl et al., 2012; Paul et al., 2017; Petrie and Yamada, 2012; Petrie and Yamada, 2016). Thus, the ability to undergo MAT helps CCs to migrate more efficiently in variable environmental conditions.

Moreover, the same triggers in different cells can stimulate different types of movement (Gao et al., 2014; Cantelli et al., 2015). For example, TGF-beta is a classical trigger of EMT, and treatment with TGF-beta led to activation of cell motility using the mesenchymal type of migration. However, Gao et al. showed that all TGF-beta isoforms stimulated collective tumor cell migration of ovarian cancer by inducing N-cadherin expression as well as by retaining epithelial shape and E-cadherin expression (Gao et al., 2014). Meanwhile in experiments with melanoma cells, TGF-beta stimulated cells to acquire rounded blebbing morphology by controlling Smad2 and CITED1-mediated contractile forces (Cantelli et al., 2015). This suggests that the ability to migratory plasticity is determined mainly by the internal characteristics of the cells. However, it is still unclear what these characteristics are and whether there are markers that will allow predicting in advance the ability of cells to migrate by one or another mode, or to obtain migratory plasticity.

## 3 Enhancement of metastasis as a side-effect of widely used anti-cancer approaches

There are increasing evidences that the use of certain therapeutic approaches to treat tumors can lead to unpredictable side effects expressed in changes in the migration potential of tumor cells, leading to the activation of metastasis and ultimately the death of the patient. Let us consider some cases of such unpredictable complications in the treatment process.

Radiation therapy is one of the main and longtime used approaches to treat cancer. It has been also shown that radiotherapy directed to destroy the tumor can cause an increase in the migratory potential of CCs (Fujita et al., 2015; Vilalta et al., 2016; von Essen, 1991) and stimulate metastasis. The activation of the EMT program and increased expression of MMPs, leading to cell spreading by the mesenchymal mechanism, are thought to be responsible for the increased migratory activity induced by radiotherapy. To prevent such complications as increased metastasis it was proposed to combine the use of radiotherapy with MMPs inhibitors. However, other studies have shown that radiotherapy under certain conditions can lead to the transition of cells to amoeboid movement (Facoetti et al., 2018), which is independent of the activity of MMPs (Fujita et al., 2007; Fujita et al., 2011; Moncharmont et al., 2014). Thus, the prevention of radiotherapy-induced metastasis appears to be a more difficult challenge that still needs to be solved. Significant up-regulation of tumoral hypoxia inducible factor-1 (HIF-1) activity as a result of radiation-induced reoxygenation was determined as a molecular mechanism stimulating migration in response to ionizing radiation (Burrows et al., 2013). Radiation also promotes the release of cytokines and growth factors by tumor cells or tumor associated fibroblasts through the induction of a damage response (Reviewed in Fujita et al., 2015).

Breast cancer pathogenesis is associated with hormone activated estrogen receptor alpha (ERα). Selective estrogen receptor modulators such as tamoxifen are widely used to stop aberrant breast cancer cell proliferation and the growth of ERα-positive breast tumors. However, tamoxifen treatment has recently been shown to increase the number of ERα-negative cancer cells that exhibit amoeboid motility, resulting in ERα-negative metastatic recurrence (Li et al., 2001; Gao et al., 2017).

Increased migratory abilities of tumor cells could also be a side effect in the case when angiogenesis inhibitors targeting the VEGF pathway are used as antitumor drugs. The action of these agents is based on inhibition of vessels’ growth in tumors and thus the limitation of nutrition for tumor cells followed by their death. VEGF-neutralizing antibody (bevacizumab) and VEGF receptor tyrosine kinase inhibitors (RTKIs) (sorafenib and sunitinib) are used for anticancer treatment as clinically validated drugs to target VEGF or its receptors (particularly VEGFR2) (Folkman, 2007). However, practical observations by сlinicians and some experimental works have shown that although the use of such agents leads to a reduction in the size of the primary tumor, it often does not prolong the survival of cancer patients for more than several months, in particular, because the treatment is accompanied by increased metastasis (Ebos et al., 2009; Loges et al., 2009; Pàez-Ribes et al., 2009).

The occurrence of side effects in the case of anti-angiogenic drugs can be explained primarily by increased hypoxia (Pennacchietti et al., 2003) caused by the lack of blood supply in the tumor (Büchler et al., 2004; Ebos et al., 2009; Loges et al., 2009; Pàez-Ribes et al., 2009). It was shown in many works that hypoxia activates EMT (Imai et al., 2003; Yang et al., 2008) and thus can stimulate metastasis through the mesenchymal motility mode (Brahimi-Horn et al., 2007; Sullivan and Graham, 2007; Yang et al., 2008), but it can also stimulate the transition to amoeboid movement, which is characterized by faster cell dissemination and lack of dependence on the activity of MMPs, which in turn further enhances the metastatic potential of tumors (Te Boekhorst et al., 2022). In particular, hypoxia has been shown to strengthen the migration of mouse breast CCs by enhancing the EMT and/or MAT of some cells. Even fully epithelial but only locally invasive UT-SCC38 human head and neck squamous carcinoma cells isolated from a patient without metastatic disease turned to a collective amoeboid transition in a 3D collagen gel (Lehmann et al., 2017) under hypoxia. Hypoxia also increased the metastasis of these cells to the lungs after intravenous injection into recipient mice. Hypoxia has been shown to promote MAT by activation of HIF1 (Lehmann et al., 2017; Te Boekhorst et al., 2022), which coordinately activates RhoA and ROCK1 expression and signaling in breast cancer cells (Gilkes et al., 2014; Leong and Chambers, 2014). Rho-dependent enhancement of contractility is a recognized trigger for the transition to amoeboid movement. The other cellular mechanisms to explain the trigger effect of hypoxia on amoeboid movement also were suggested. They are the reduction of MT1-MMP (matrix metalloproteinase 14) activity (Lehmann et al., 2017), or disruption with cysteine protease calpain-2, which cleaves the focal adhesion adapter talin-1, limits beta 1 integrin activity, and strongly enhances metastasis (Te Boekhorst et al., 2022).

Another example of increased metastasis as a side effect of well-known and widely used anticancer drugs that destroy microtubules - vinca alkaloids (vincristine, vinblastine, *etc.*) or drugs that disrupt microtubular dynamics (taxols, taxanes). Their antitumor effect is since these substances are mitotic poisons, i.e., they disrupt normal microtubule functioning, which also leads to the destruction of the mitotic spindle resulting in cell division arrest. Although the role of microtubules in the regulation of cell motility has been known for a long time (Rodionov et al., 1993; Waterman-Storer et al., 1999; Rodriguez et al., 2003; Etienne-Manneville, 2004), primarily their antimitotic activity is the main reason for the development of drugs on the base of antimicrotubular agents (Škubník et al., 2020). It has been recently shown that in some cases, the use of antitubulins can lead to increased metastasis. Thus vincristine increased ability of human colorectal cancer HCT116 cells to migrate through the filters in the Transwell assay (Jin et al., 2016) and stimulate amoeboid-like motility of MKN45 cells by activating RhoA/ROCK/MLC signaling pathway (Eitaki et al., 2012). In another work, it was shown that the long time use of paclitaxel in experiments with A2780 and OAW42 human epithelial ovarian cancer cells resulted in arising of drug resistant cells whose migration rate was significantly increased. A protease-independent amoeboid mode of migration was described for these cells (Kapoor et al., 2018). It was shown also that in some experimental systems depolymerization of microtubules leads to the development of membrane blebs (Keller and Eggli, 1998; Sugiyama et al., 2015), which is due to release of GEF-H1 from microtubules after microtubule depolymerization, and leads to increase in acto-myosin contractility by activation of GEF-H1/RhoA/ROCK/MLC2 signaling (Krendel et al., 2002; Rafiq et al., 2019). Antimicrotubular agents are widely used in anticancer treatment, but their effect is not selective to CCs and most of them are quite toxic, since their action is directed to all dividing cells. The active search for new agents or new combinations of antitubulins with other agents being developed to reduce the toxic effect is in progress (Škubník et al., 2020). Several reviews have been published describing the principles and mechanisms of antitubulins’ action (Dumontet and Jordan, 2010; Škubník et al., 2020) and the possibility of their use in the treatment of malignant diseases, when combining them with other agents. However, when developing new treatment approaches we should keep in mind that under certain conditions antitubulins can stimulate the activation of metastasis due to the transition to amoeboid movement.

Since increased migration is an obvious sign of an aggressively developing tumor, one of the approaches to treatment is the search for agents capable of stopping migration of cancer cells. The increased expression of matrix metalloproteases, which degrade extracellular matrix and thus facilitate tumor cell migrationis considered one of the most obvious and long-noticed changes associated with tumor development that contributes to activation of tumor cell motility by the mesenchymal mechanism after EMT (Egeblad and Werb, 2002; Overall and Kleifeld, 2006). The first attempts to stop tumor cell migration and consequently slow down or stop the formation of metastases by using MMPs inhibitors were made almost 30 years ago. However, this approach did not bring the expected result, and in some cases the dissemination rate of tumor cells even increased (Zucker et al., 2000; Coussens et al., 2002; Overall and López-Otín, 2002; Wyckoff et al., 2006; Tu et al., 2008; Yan et al., 2016). One of the reasons for this failure turned out to be the ability of tumor cells under MMPs inhibition to change their migration mode to an amoeboid one, which is independent of MMPs activity and often characterized by faster cell migration (Wolf et al., 2003a, 2003b; Yan et al., 2016). Studying of molecular mechanisms stimulating MAT under inhibition of proteolytic activity showed that the main regulator in this case is an increase in Rho-ROCK pathway activity (Sahai and Marshall, 2003; Rösel et al., 2008; Fagan-Solis et al., 2013).

In Table 1 we summarized the main cases of induction of metastatic tumor potential under the influence of different therapeutic treatments. Thus, it becomes clear that it is very important to take into account the possibilities of migratory plasticity while researching the effect of a particular agent on tumor growth and progression, and especially when developing new treatment approaches.

**TABLE 1:** Examples of the induction of metastatic tumor potential under the influence of different therapeutic treatments.

Applied treatment	Tumor/Cells	Expected effect	Undesirable side effect	Possible mechanisms	References
Radiotherapy	Tumors of different origin	Tumor cell death through the induction of irreparable DNA damage and cell cycle arrest	Recruitment of circulating tumor cells (CTC); enhanced invasion/metastasis	Disruption of cancer cells and cells from microenvironment led to	Reviewed in: von Essen, (1991), Moncharmont et al., 2014, Martin et al., 2017; , La Verde et al. (2021)
				Activation EMT	
				Hypoxia •	
				Activation of intracellular signal pathways and secretion of MMPs	
				Reorganization of microenvironment and tumor vasculature	
Examples	Mouse mammary carcinoma	Destruction of tumor by local irradiation	Spreading to lymph nodes and formation of lung metastasis	???	Kaplan & Murphy, (1949)
	Glioblastoma multiforme (GBM)	Analysis of influence of irradiation on cell motility behavior	Increase in tumor cells invasiveness	Activation of Rho signaling *via* PI-3K	Zhai et al. (2006)
	Tongue squamous cell carcinoma (TSCC) cells	*In vitro* experiments (wound healing, transwell migration, Matrigel invasion assays) estimation of migratory features	Dose-dependent increase of invasive and migratory potentials of cells	Altering biomechanical properties of cells and the organization of the actin cytoskeleton	Zheng, et al. (2015)
	Lewis lung carcinoma in mice, subcutaneous injection	Study the influence of radiation therapy on metastasis formation	Significant increase in the number of lung metastasis	Growth of previously dormant lung metastases	Camphausen et al. (2001)
	Lewis lung carcinoma	Formation of lung metastasis *in vivo* and invasion assays *in vitro* (migration through Matrigel-coated Boyden chamber and chicken chorioallantoic membrane)	Significant increase in cell invasiveness and lung metastasis formation	Induction of MMP-9 at both the transcriptional and translational levels	Chou et al. (2012)
	Human pancreatic cancer line, MIAPaCa-2	Studying of migration and invasion in *vitro* experiments	Increase in cell motility and invasiveness	Induction of matrix metalloproteinases (MMP) under X-ray or γ-ray irradiation, acquisition of migration plasticity	Fujita et al. (2011)
Surgical intervention at different levels: (Biopsies and other diagnostic procedures; radical resection of primary tumor, resection of tumor-bearing lymph nodes)	Tumors of different origin	Diagnostic procedure, Tumor resection, removal of metastatic lesions	Stimulation of CTC formation, multiple metastasis along needle path; Spontaneous metastasis in the lungs, liver, and lymph nodes	Release of growth factors and inflammation in result of destruction of cells	Reviewed in Martin et al. (2017), Tohme et al. (2017), Man et al. (2007), Zheng et al. (2018)
Chemotherapy	Tumors of different origin	Suppression of tumor cell proliferation, death of tumor cells and tumor growth halting	Stimulation of invasion and metastasis, survival time decrease	Selection/generation of disseminating CSCs, mobilization and recruitment of pro-tumorigenic immune cell subsets, contribution to generation of pro-metastatic niches favoring extravasation, cell migration capacities stimulation (EMT and MAT)	Reviews:, D'Alterio (2020), Karagiannis et al. (2018), Martin et al. (2017)
MMPs inhibitors: Batimastat, Marimastat Ilomastat	Glioblastoma, ovarian cancer, small cell lung cancer; Glioblastoma cells	Inhibiting of metastasis and decelerating of tumor development (predicted based on the preclinical studies)	Multiple side effects and often increase in metastasis formation	MAT through RhoA/Rho kinase (ROCK)/myosin light chain (MLC) pathway	Coussens et al. (2002), Hidalgo and Eckhardt, (2001), Bramhall et al. (2001), Yan et al. (2016)
Inhibition of cell-ECM interactions Anti-integrin antibodies: vitaxin, etaracizumab, intetumumab (CNTO95); or abituzumab Volociximab	Advanced metastatic melanoma; androgen-independent prostate cancer; renal cancer; melanoma, sarcoma, colorectal, epithelial ovarian or primary peritoneal cancer, advanced non-small-cell lung cancer (NSCLC)	Halting of metastasis and death of tumor cells due to disruption of regulatory pathways; inhibition of angiogenesis	Increase in cancer cell invasiveness and metastasis Or failed to show benefit	The stem/progenitor cell recruitment; activation of the ERK1/2 and p38 MAPKs signaling cascades	Alday-Parejo (2019), Vellón et al. (2010), Posey et al. (2001)
Angiogenesis inhibitors: anti-VEGFR2 antibody (DC101), Receptor tyrosine kinase inhibitors, (sunitinib and sorafenib)	Pancreatic neuroendocrine cancer; orthotopic models of 231/LM2-4 tumor; GMB, carcinoma	Disruption of blood supply to the tumor, death of tumor cells	Accelerate metastatic tumor growth and decrease in overall survival (in mice)	Hypoxia, EMT, MAT	Pàez-Ribes et al. (2009), Ebos et al. (2009), Li et al. (2021), Reviewed in Loges et al. (2009)
Vincristine (in high concentration)	Human gastric adenocarcinoma MKN45 cells; human lung adenocarcinoma; A549 cells; human colorectal cancer cells HCT116	Death of proliferated cells, tumor reduction	Increase in metastasis, stimulation of MAT and migration through the filters in the transwell assay	Activations of GEF-H1/RhoA/ROCK/MLC signaling	Eitaki et al. (2012), Jin et al. (2016)
Doxorubicin	Metastatic breast cancer	Tumor growth halting	Lung and bone metastasis of breast cancer cells in orthotopic xenograft model	Stimulation of EMT by activation of TGFb pathway	Bandyopadhyay et al. (2010)
Hormone therapy Tamoxifen	ERα-positive breast cancer	Halting of aberrant proliferation of ERα-positive breast cancer cells	Increased risk of ERα-negative contralateral tumors	Loss of ERα leads tocell amoeboid-like movement by vinculin suppression	Li et al. (2001), Gao et al. (2017)

Cell migration using any of the presented migratory modes is based on cytoskeleton reorganization. Thus, the altered expression of cytoskeleton proteins or changes in signaling pathways regulating cytoskeleton reorganizations can serve as either targets for inhibiting metastasis or markers for prediction of possibility of appearence of migratory plasticity as a possible undesired effect of applied treatment. Thanks to the development of proteomics and next generation sequencing (NGS) methods, many new data on the alterations in expression of various proteins involved in cytoskeleton reorganization in different tumors have been recently obtained.

However, little is known about what features of the cytoskeleton can determine the possibility of transition from one motility mode to another. This question seems to be very important, because if we can assess in advance the risk of migratory plasticity and the possibility of inhibiting transitions when planning tumor treatment and prescribing certain drug regimens, it will allow us to avoid complications associated with the development of increased metastasis.

## 4 The selection of individual cytoskeleton proteins as drug targets and the emergent problems associated with migratory plasticity

### 4.1 Actin and actin-binding proteins

The reorganization of the actin cytoskeleton is the main cellular machinery that underlies cell motility, so there have been many attempts to stop the dissemination of tumor cells using small molecules directly acting either on actin or its dynamics. These are compounds from the anti-actin drug groups which either destabilize actin filaments (cytochalasins, geodiamolides, and latrunculins) or disrupt actin dynamics (jasplakinolide, chondramide, and cucurbitacin E) (for reviews see Bijman et al., 2008; Bonello et al., 2009; Fenteany and Zhu, 2003; Gandalovičová et al., 2017). For many of these compounds the ability to stop the migration and invasion of various tumor cells in vitro experiments was shown. But, unfortunately since the actin reorganization is involved in many vital processes, most of anti-actin drugs have shown intolerable toxicity to be used as anti-tumor agents *in vivo*, in particularly because of very high possibilities to impact cardiovascular functions. Since the mechanisms regulating actin reorganization are now well understood, this opens up the possibility to consider proteins that interact with actin and participate in the more subtle regulation of actin cytoskeleton dynamics as drug targets (Fu et al., 2018; Aseervatham, 2020). We will observe the possibilities to use the proteins regulating actin dynamics as promising drug target and will discuss problems, which could arise because of involvement of these proteins into the regulation of migratory plasticity.

### 4.2 Regulators of actin dynamics

#### 4.2.1 Arp2/3 complex

One of the main participants in polymerization of actin filaments, responsible for cell movement by the mesenchymal mode of migration, is the Arp2/3 complex consisting of seven proteins, which regulates the formation of branching actin network (Mullins et al., 1998; Svitkina and Borisy, 1999). By now, the cellular mechanisms of Arp2/3 involvement in actin polymerization and the signaling pathways regulating them are well understood (Suraneni et al., 2012; Svitkina, 2018; Gautreau et al., 2022). Numerous studies have shown that Arp2/3 expression is enhanced during the progression of many tumors (although not in all cases) (Iwaya et al., 2007; Otsubo et al., 2004; Steinestel et al., 2015; reviewed in Molinie and Gautreau, 2018). In particular, high Arp2 expression often correlates with aggressive behavior and poor prognosis (Otsubo et al., 2004; Semba et al., 2006; Zheng et al., 2008; Sun et al., 2014). Therefore, a lot of studies are aimed at finding specific Arp2/3 inhibitors that could be used as antitumor agents (Yoon et al., 2019). Small molecules CK666 and CK869 were chemically synthesized, their action is based on disruption of interactions between Arp2 and Arp3 subunits (Hetrick et al., 2013; Henson et al., 2015). These agents inhibit the formation of lamellipodia and stop the migration and invasion of tumor cells (Liu et al., 2013; Zhou et al., 2016). Moreover, there are some drugs that are already approved by FDA (US Food and Drug Administration) and are used in the clinic for purposes other than antitumor therapy, but for which Arp2/3 inhibition have recently been shown as a new function. One such compound is benproperine, discovered through a Connectivity Map (CMap)-based drug discovery strategy (a new screening method) and usually used as antitussive drug. Benpropenine selectively inhibits tumor cell motility through interaction with the ARPC2 subunit and has no effect on normal cells. Its anti-metastatic activity was confirmed in *vivo* mouse model (Choi et al., 2019; Yoon et al., 2019). Another agent that also affects ARPC2 is pimozide - which belongs to the diphenylbutylpiperidine class of drugs and targets dopamine receptor D2 (DRD2) by reducing dopamine activity. This compound is also FDA-approved as an antipsychotic drug. Pimozide decelerates Arp2/3 - dependent actin polymerization and inhibits the vinculin mediated recruitment of ARPC2 to focal adhesions in cancer cells (Choi et al., 2019). One more agent Polo like kinase 4 (Plk4), which is an upstream effector of actin nucleating activity of the Arp2/3 complex has been suggested as a therapeutic target (Kazazian et al., 2017). Plk4 depletion suppressed cancer invasion and induced an epithelial phenotype in poorly differentiated breast cancer cells. Thus using of agents affecting Arp2/3 activity as drugs to stop metastasis looks very tempting. For more details on the main Arp2/3 inhibitors and their potential use as anti-tumor agents see review (Chánez-Paredes et al., 2019). However, one must keep in mind that numerous studies show that suppression of actin polymerization in particular by inhibiting the action of the Arp2/3 complex stimulates the transition to amoeboid movement in cells of different types (Beckham et al., 2014; Bergert et al., 2012; Chikina et al., 2019a,b; Derivery et al., 2008; Logue et al., 2018; Obeidy et al., 2020). The authors of these studies suggest the weakening of the connection between the membrane and the submembrane actin cortex, or disruption of the regularity of this cortex, as the main triggers that stimulate MAP in cases of Arp2/3 suppression. The other explanation of triggering the transition to amoeboid motility by Arp2/3 inhibition could base on some evidences that the change in Arp2/3 activity lead to alteration of actin-myosin contractility. It was shown that inhibition of Arp2/3 lead to rearrangement of branched actin filaments in lamellipodia and reorganization of them to antiparallel arrays of long actin filaments that were able to recruit non muscle myosin II and thus became contractile (Henson et al., 2015). Also RNAi-mediated knockdown of the Arp2/3 complex in neuroblastoma cells led to activation of RhoA (Korobova and Svitkina, 2008) and CK666 inhibition of Arp2/3 activity in melanoma cells led to increase of cortex tension and intracellular pressure (Logue et al., 2018). But, according the other research, inhibition of the Arp2/3 complex in mouse embryo fibroblasts led to a reduction of both RhoA activity and cell contractility (Huang et al., 2019). Regardless of the mechanisms underlying the activation of amoeboid movement in the case of Arp2/3 inhibition, this possibility must be taken into account when developing new therapeutic approaches to treat metastasis development.

#### 4.2.2 ADP/cofilin

Other regulators of actin dynamics are actin depolymerizing factor (ADP)/cofilins, which contribute to cytoskeleton dynamics by promoting rapid disassembly of actin filaments and thereby restoring the G-actin pool for subsequent polymerization (Plastino and Blanchoin, 2018). They play essential role in regulation of mesenchymal cell migration and alterations in their activities, which contribute to manifestation of malignant phenotype and migration behavior of cancer cells (Shishkin et al., 2016). Recent studies revealed the significance of ADF/cofilins for amoeboid motility. Particularly for fast amoeboid motility of melanoma cells occurred as leader bleb-based migration (LBBM), cofilin-1 and ADF were identified as essential factors required for migration (Ullo and Logue, 2021). ADF and cofilin-1 show additive effect and, together they optimize actin disassembly and myosin contractility at the leader bleb necks and thus promote rapid cortical actin flow in leader blebs. Such actin flow in combination with non-specific friction in confinement conditions provides the driving force for fast amoeboid cell movement (Ruprecht et al., 2015). Thus, high activity of cofilin one could relate to increase the metastatic properties of tumor cells. Cofilin activity is regulated by phosphorylation/dephosphorylation (Mizuno, 2013). The main regulators are LIM kinases (LIMKs) which negatively regulate cofilin by phosphorylation at Ser 3, while reactivation is processed by protein phosphatase slingshot homologs (SSHs) (Niwa et al., 2002; Mizuno, 2013). It was shown that overexpression of SSH1 is associated with advance stages of pancreatic cancer with high metastatic potential (Wang et al., 2015). As a result of screen for new SSHs inhibitors the compound sennoside A was characterized as a novel inhibitor of SSHs and potential regulator of metastasis through the regulation of cofilin phosphorylation (Lee et al., 2017). Decrease of cofilin activity in result of sennoside A-induced SSH inhibition significantly reduce motility of pancreatic cancer cells, their invasion through matrigel, transendothelial migration through underlied HUVECs cells and liver metastasis formation in experiments with mouse model. Thus, the proteins from family of ADF/cofilins could represent novel promising anti-cancer targets.

### 4.3 Actin linker proteins

#### 4.3.1 Fascin

The proteins that cross link actin filaments and thus organize the overall architecture of the actin cytoskeleton are of great importance in the regulation of actin cytoskeleton reorganization. One of such proteins, Fascin, is a bivalent actin-binding protein that binds actin filaments together in the filopodia or invadopodia and imparts additional rigidity to actin bundles (Svitkina et al., 2003; Li et al., 2010). Fascin is practically absent in normal adult epithelial cells (Hashimoto et al., 2005; Zhang et al., 2008), but its expression is significantly enhanced in many carcinomas. Increased activity of this proteinis associated with advanced tumor progression and poor prognosis (Machesky and Li, 2010). Fascin-1 expression is associated consistently with increased risk of mortality in breast, colorectal and esophageal carcinomas, and with metastasis in colorectal and gastric carcinomas (Tan et al., 2013). Activation of Fascin expression is considered to be a part of the EMT program that contributes to increased invasion and metastasis by stabilization of actin bundling during formation of filopodia and invadopodia (Li et al., 2010). Fascin promotes invasion and metastasis of tumor cells by the mesenchymal mechanism (Machesky and Li, 2010). In addition Fascin overexpression upregulates expression and recruitment of MMPs, supporting a role for Fascin in ECM remodeling what also facilitate mesenchymal motility of cells (Al-Alwan et al., 2011). It was recently shown that in special condition (under conditions of confinement and low substrate adhesion) the increase of Fascin expression promotes the transition of melanoma and osteosarcoma cells to amoeboid migration (Clancy et al., 2019; Adams et al., 2021).

Many studies are focusing on searching for inhibitors of Fascin as agents blocking metastasis. Several compounds inhibiting activity of this protein were proposed as inhibitors of cell migration and invasion *in vitro* and blocators of metastasis in mouse models. Between them, there are Migrastatin (Chen et al., 2010) and small molecule G2 that blocks actin structures, migration, and invasion of colorectal cancer cells as Fascin-dependent (Montoro-García et al., 2020). Phase I clinical trials have begun testing a Fascin inhibitor NP-G2-044 to treat metastatic carcinomas (Alburquerque-González et al., 2020; Alburquerque-González et al., 2021). The FDA-approved anti-retroviral raltegravir (RAL) was also identified as a potential Fascin1 blocker. *In vitro* and *in vivo* experiments with colorectal cancer cells showed that RAL exhibits Fascin1-binding activity and Fascin1-dependent anti-invasive and anti-metastatic properties. It should be noted that recent studies have shown that Fascin performs many other functions in the process of oncogenesis, apart from the regulation of cancer cells migration, such as metastatic colonization, anoikis resistance, chemoresistance, and cancer cell stemness [reviewed in Lin et al. (2021)]. While Fascin is not expressed in adult epithelial tissue, it is expressed in other adult tissues (dendritic cells, endothelial cells, and cells from the mesenchymal and neural crest lineage). Because of that fact the inhibition of Fascin could led to arising of different negative side effects, such as neuronal, kidney, endocrine, wound healing, and immune defects (Lin et al., 2021). That means questions of using Fascin inhibitors as antimigratory drugs need additional investigations.

#### 4.3.2 Filamin A

Another family of actin-binding proteins that is changed during tumor progression is the family of filamins, which are scaffolding proteins and couple actin cytoskeleton to extracellular matrix. FilaminA (FLNA) plays an important role in remodeling the actin cytoskeleton and thus in cancer development and metastasis. But the data on the involvement of filamins into the regulation of tumor progression are quite contradictory (reviewed Savoy and Ghosh, 2013; Tian et al., 2013). It was recently shown that FLNA is a potential driver gene of breast metastasis. Its low expression is associated with enhancing of 5 years’ relapse survival rate by 15%, and stable FLNA knockout led to decrease of tumor cell migration and invasion. FLNA knockout could inhibit formation of local or distal metastasis in xenograft mouse experiments (Zhou et al., 2022). The authors did not find a link between FLNA knockout and changes in EMT markers, but found that it leads to a decrease in MMP-1 expression, and therefore conclude that FLNA might intervene in metastasis *via* the regulation of MMP-1 expression. In the other work it was shown that FLNA levels were increased in malignant breast or brain tissues, but not in normal control tissues (Savoy and Ghosh, 2013). However, another study showed that FLNА down-regulation stimulates migration and invasion of breast cancer cells *in vitro*, and promotes metastasis formation in xenograft breast cancer mouse models (Xu et al., 2010). The authors showed that FLNA regulates disassembly of focal adhesions *via* a calpain-dependent mechanism and thus inhibits cell migration. This seeming contradiction could be explained by the fact that disruption of focal adhesions prevents cell migration by a mesenchymal mechanism, but can serve as a trigger for the transition to amoeboid movement. It was recently shown that an increase in FLNA expression promotes the transition to amoeboid movement driven by leader bleb (Leader Bleb-Based Migration - LBBM) of melanoma cells under conditions of low adhesion and confinement (Adams et al., 2021). Suppression of FLNA with siRNA significantly reduces the number of cells able to switch to this mode of migration. Using an atomic force microscope it was shown that FLNA, as well as Fascin, promotes cortical tension and intracellular pressure. Plasma FLNA was suggested to be a specific and sensitive diagnostic marker for patients with high-grade astrocytoma or metastatic breast cancer (Alper et al., 2009).

#### 4.3.3 Eps8

It has also been shown that other less studied actin linkers are also important in determining the modes of cancer cells migration. Epidermal growth factor receptor pathway substrate 8 (Eps8) is an actin bundling and capping protein whose capping activity is inhibited by Erk. Erk-MAP kinase pathway is activated in tumor progression. Upregulation of Eps8 in cancers correlates with invasivity and poor prognosis (Griffith et al., 2006; Wang et al., 2009; Kang et al., 2012). Recent studies have shown that, increase of Eps8 expression contribute to the transition of melanoma cells migrating under confinement conditions with low adhesion, to amoeboid LBBM movement (Logue et al., 2015). This motility mode has been described under *in vitro* conditions, but LBBM morphology has also been observed for melanoma cells migrating in the dermis of living mice (Tozluoglu et al., 2013; Charras and Sahai, 2014).

#### 4.3.4 Actinins

The other family of actin-bindings proteins involved in remodeling of actin cytoskeleton and regulation of cell motility are alpha actinins (ACTN) which are actin cross-linking proteins that belong to the spectrin superfamily. They are found in all cell types. In mammary epithelial cells, the increased α-actinin-1 (ACTN1) level promotes cell migration and induces scattering of acini-like structures in Matrigel. The possible reason for this is the increased ACTN1 expression leads to destabilization of E-cadherin-based adhesions and thus promotes the migratory potential of breast cancer cells. In case of basal-like breast cancer the increased expression of ACTN1 contributes to the transition to amoeboid movement (Kovac et al., 2018).

The important role in migratory plasticity and cancer progression was shown for other member of ACTN family - α-actinin 4 (ACTN4), which is also able to interact with plasma membrane lipids and integrins (Foley and Young, 2014; Liem, 2016). It was shown that ACTN4 has essential rolefor metastasis development (Honda et al., 2005; Kikuchi et al., 2008; Tentler et al., 2019). Its expression enhanced in breast carcinoma, colorectal cancer, nonsmall-cell lung cancer, pancreatic cancer, and ovarian cancer. It was considered as a diagnostic marker for ovarian cancer (Yamamoto et al., 2009). Expression of ACTN4 was significantly upregulated in the melanoma invaded regions of the skin dermis, as compared with non-invaded skin areas (Shao et al., 2014). ACTN4 was significantly increased in the castration-resistant prostate cancer patients (CRPC) compared to the patients well-controlled with primary androgen deprivation therapy and thus was suggested as potential therapeutic target for CRPC (Ishizuya et al., 2020). In the other study it was shown that ACTN4 plays an important role in maintaining the amoeboid morphology of invasive melanoma, and thus promotes dissemination through collagen-rich matrices. Knockdown of ACTN4 in highly metastatic amoeboid melanoma WM1158 cells was found to lead to a switch from amoeboid to mesenchymal mode of motility (Shao et al., 2014).

#### 4.3.5 Tropomyosins

Other important regulators of the architecture and dynamics of the actin cytoskeleton are tropomyosins (Tpm). Tpm is a family of actin-binding proteins consisting of six isoforms which divided into high molecular weight (HMW) or low molecular weight (LMW) tropomyosins. Downregulation of HMW-specific Tpm isoforms is a common characteristic of transformed cells that leads to increase of migration and invasion of cancer cells (Zheng et al., 2008). Conversely, elevated expression of a LMW Tpm3.1 isoform (former name is Tm5NM1) has been shown for transformed rat fibroblasts, and highly metastatic melanoma; and elevated expression of Tm4 is associated with lymph node metastasis in breast cancer [reviewed in Gunning et al. (2008)]. The anti-tropomyosin drugs TR100 and ATM3507 targeted at Tpm3.1 which assumed to stop cancer cells migration have been developed (Stehn et al., 2013). Their action is based on disruption of the interaction between tropomyosin and actin and they show anticancer properties in melanoma and neuroblastoma cells *in vitro*. Since these compounds act mainly on tumor cells and work at the level of actin network organization, they inhibit both mesenchymal and amoeboid cell movement, therefore the authors consider them a good candidate for inhibiting tumor cell dissemination. The influence of different HMW isoforms of Tmp on motility of cancer cells is rather complicate. It was shown that downregulation of Tpm2.1 increases the rate of amoeboid cell migration and invasion in experiments with breast cells MCF7 (Shin et al., 2017). This can be explained by the fact that Tpm2.1 modulates in actomyosin contractility mediated by Rho-ROCK that is essential in cell migration. It was also shown earlier that loss of Tpm2.1 in colorectal cancer cell line HS675T upregulated the levels of active RhoA.

Altogether it means that attempts to stop or slow down cancer cell dissemination by acting on actin-linker proteins carry the potential risk of stimulating migratory plasticity.

### 4.4 How do actin linkers affect the choice of motility modes?

Several possible mechanisms for the involvement of actin linkers in the increase in cell migration potential have been considered. An increase in the expression of fascin is associated with significant increase in the number of filopodia (Vignjevic et al., 2006). In this regard, the hypothesis has been proposed that the increased expression of fascin precisely due to the multiplicity of filopodia contributes to the interaction with ECM and increased explorative movement of cancer cells, and hence metastasis. The association of enhanced filopodia formation with blebbing has been shown in several works (Yoshida and Soldati, 2006; Ma and Baumgartner, 2013; Tyson et al., 2014; Meyen et al., 2015; Goudarzi et al., 2017). It also directly has been shown that the transition from mesenchymal to amoeboid movement occurs through the stage of formation of multiple filopodia (Chikina et al., 2019b). We hypothesized that the presence of a large number of filopodia may facilitate the transition to amoeboid motility because the uniform organization of the submembrane actin cytoskeleton is disrupted at the base of the filopodia by the formation of gaps in a dense actin network (Chikina et al., 2019b). It was suggested earlier that bleb formation is enhanced by local weaknesses in the actin cortex and by locally reduced interactions of the cortex with the plasma membrane (Paluch and Raz, 2013). Thus, disruption of actin cortex integrity caused either by actin network damage by a low concentration of Latrunculin A (Keller, 2000) or mechanical local cortex disruption by lasar ablation (Tinevez et al., 2009; Sedzinski et al., 2011), as well as sparsityof actin network at the base of filopodia could be triggers that provoke blebbing (Alexandrova et al., 2020).

The importance of actin-binding proteins for amoeboid motion is probably explained by their participation in the organization of the actin cortex architecture, which in turn maintains the necessary cortical tension and intracellular pressure suggesting a general role for cross-linking in the mediation of these basic physical requirements for bleb formation (Tinevez et al., 2009; Clark et al., 2013; Logue et al., 2015; Wang et al., 2016; Koenderink and Paluch, 2018; Adams et al., 2021).

## 5 Non-actin cytoskeletal regulators of migratory plasticity

### 5.1 Focal adhesions

The system of cell adhesions with the extracellular matrix is closely related to the actin cytoskeleton. Since the efficiency of cell migration by the mesenchymal mechanism depends on the ability of cells to form focal adhesions (FA) with ECM, one of the ways to suppress the migration activity of cells is to suppress the ability to form such structures. Integrins are the transmembrane receptors which provide connections between actin cytoskeleton and ECM (Gauthier and Roca-Cusachs, 2018). In the past few years, pre-clinical assays have revealed that integrin targeted therapy, including mAbs and synthetic molecules, strongly imparts antitumor effects (Aksorn and Chanvorachote, 2019; Alday-Parejo et al., 2019). Several integrin antagonists for anticancer therapy are currently developed and are at the different stages of clinical trials. For example Vitaxin1, a humanized monoclonal antibody that blocks human and rabbit αvβ3 integrins, is in clinical trials for metastatic melanoma, including high grade cancer, and prostate cancer (Tucker, 2006; Gramoun et al., 2007). Bevacizumab (Avastin, LM609, Genentech) which is a mouse anti-human integrin αvβ3 is in trial to treat glioblastoma, metastatic melanoma, renal cell carcinoma and non-small cells lung cancer (NSCLC) (Kuwada, 2007; Mas-Moruno et al., 2010). However, clinical assays have not confirmed the anti-tumor effects obtained in *vitro* experiments. It has been revealed that Avastin treatment may increase the number of cancer stem cells (CSCs) in breast cancer (Conley et al., 2012). The failure of effect of integrin treatment in relation to patient survival time and occurrence of metastasis may be results of the complexity of integrin mechanisms, the development of resistance to anoikis, and the ability of different integrins to compensate each other (Li et al., 2021). Also it is need to take in account that the decline in the formation of adhesion contacts with the substrate is one of the factors that cause the switch to amoeboid movement, regardless of whether the suppression of adhesions formation is caused by reduced adhesiveness of the matrix (Bergert et al., 2012; Liu et al., 2015), or blocking the integrin-ECM interactions (Carragher et al., 2006), or failure of other components of focal adhesions. For example, suppression of vinculin in breast cancer cells MCF-7 stimulates the switch of these cells to amoeboid movement *in vitro* and increases the number of metastasis in *vivo* experiments in a xenograft mouse model (Gao et al., 2017). Depletion of talin1 (the protein which directly link integrin to actin filaments) in murine megakaryocytes resulted in strong membrane blebbing (Wang et al., 2008). Low adhesiveness of the substrate combined with confinement are conditions for the transition of the cells to amoeboid motility (Liu et al., 2015; Logue et al., 2015; Yip et al., 2015).

It has recently been shown that increased metastatic activity as a side effect of tamoxifen treatment of ERα-positive breast cancer is due to the fact that decreased ERα expression may promote amoeboid migration breast cancer cells because ERα suppresses amoeboid cell movement by upregulating vinculin (Gao et al., 2017). The regulation of FA dynamics is also essential for determination of motility mode. The important regulators of FA dynamics are the components of FA prototypical oncogene kinase Src and focal adhesion kinase (FAK). FAK is commonly overexpressed in cancer and has been considered a high value druggable target for therapy (reviewed in Dawson et al., 2021; Chuang et al., 2022). There are some evidences that active FAK facilitates 3D matrix invasion through increased cellular stiffness and transmission of actomyosin-dependent contractile force in dense 3D extracellular matrices (Mierke et al., 2017). The possibility to use FAK as antitumor target is widely studied and two FAK inhibitors IN10018 (other name is BI 853520) (Hirt et al., 2018) and Defactinib (VS-6063; PF-04554878) (Klaeger et al., 2017) are currently under clinical evaluation in a number of phase I/II combination trials. But there are some evidences that inhibition of FAK could stimulate metastasis (Batista et al., 2014). It was shown that the inhibition of FAK in human embryonic stem cells prolonged non-apoptotic blebbing (Weng et al., 2018) and that one of the partners of FAK involved in regulation of focal adhesion dynamics RhoA guanine nucleotide exchange factor (GEF) Net1 controls amoeboid motility during invasion of extracellular matrix (ECM) (Carr et al., 2013). The other kinase Src phosphorylates and thus activates FAK. Activation of Src kinase is frequently observed in malignant cells (Guarino, 2010) and is essential for invasion and other tumor progression-related events such as epithelial-to-mesenchymal transition (EMT) and development of metastasis. In recent works it was shown (Logue et al., 2018; Ullo and Logue, 2018) that Src inhibitor Dasatinib, approved by FDA for the treatment of chronic myeloid leukemia, reduced focal adhesions, led to the reduction of cortex actin density and provoked blebbing in melanoma A375 cells. By atomic forse microscopy it was revealed that Dasatinib reduced cortical tension and intracellular pressure. But in conditions of confinement melanoma cells demonstrated amoeboid leader bleb-based migration (LBBM) which was unsensitive to either Dasatinib treatment or to downregulation of Src activity by expression of dominant negative Src^K295R^. It gives the ability to speculate that the essential point in switching to amoeboid motility mode is combination of weak cortical actin and increased intracellular pressure, which could be induced either by intracellular regulations, or by environmental conditions (motility in confinement). The tyrosine kinase Src could be a signal transducer that can potentially influence transitions between migration modes. Thus, a change in the microenvironment during cancer cell migration, in particular exposure to confined conditions, can stimulate the transition to an amoeboid mode of motility for cells with altered regulation under the influence of drug treatment. When developing new anticancer approaches, we cannot avoid the influence of microenvironment on cancer cells, but we must try to predict the emergence of new properties of these cells that allow for migratory transitions.

### 5.2 Vimentin

Another cytoskeletal system involved in the regulation of cell motility and undergoing significant changes in the process of tumor progression is the system of intermediate filaments (IF). In the process of EMT, the set of IF changes and keratin IF typical for epithelial cells are replaced by vimentin IF (VIF) which are characteristic of motile mesenchymal cells (Kokkinos et al., 2007). The appearance of vimentin is considered as one of the main markers of disseminating tumor cells (Sun et al., 2010; Satelli and Li, 2011; Chung et al., 2013; Wei et al., 2019). Increased vimentin expression is associated with increased metastasis and poor prognosis in various cancers, such as colorectal cancer (Du et al., 2018), oral squamous cell carcinoma (Liu et al., 2016). At the same time, vimentin is not only marker of EMT and can itself play different roles in increasing the metastatic potential of cancer cells (Sutoh Yoneyama et al., 2014; Patteson et al., 2019b). VIF stimulate directed mesenchymal migration, regulate focal adhesion formation (Gregor et al., 2014), it is essential for invadopodia formation (the specific protrusions which are driving invasion) (Schoumacher et al., 2010). This is noteworthy a significant importance of VIF for mechanical protection of nuclei from destruction during invasion in the limited spaces is also shown (Sutoh Yoneyama et al., 2014; Castro-Castro et al., 2016). Moreover, expression of vimentin itself stimulates directional cell movement and enhances metastasis of tumors of different origin (Wei et al., 2008; Vuoriluoto et al., 2011; Liu et al., 2016). Therefore, it is not surprising that disruption of the VIF system in the cell has been proposed as one of the approaches to stop the dissemination of tumor cells. But development drugs based on specific vimentin suppression is complicated by the fact that there are almost no small molecules that cause disruption of VIF. However, there are approaches to disrupt the dynamics distribution of vimentin, leading to its collapse or preventing its assembly into complete mature filaments [Reviewed in Strouhalova et al. (2020)]. The one of proposed antitumor agents, whose action is targeted to vimentin, is witaferin A, which initiates perinuclear collapse of VIF and acts both on cell migration and proliferation, disrupting the dynamics of mitosis and causing apoptosis. Beside that the use of aptamers, which are single-stranded short RNA or DNA molecules that bind to specific peptides, providing a highly specific effect is a new approach (Marshall and Wagstaff, 2020). In particular, as an example of an antivimentin drug, it is proposed to use the RNA aptamer P15 which binds vimentin on the cell surface and specifically targets pancreatic adenocarcinoma cells. It is internalized by the cells and inhibits migration while having no effect on cell proliferation (Yoon et al., 2017).

The potential of using of vimentin as anti-cancer target is rather widely discussed, but recently it was shown that loss of vimentin increases cell motility through constricting spaces and small pores (Patteson et al., 2019a). In melanoma cells RNAi-mediated depletion of vimentin increases amoeboid leader bleb-based migration (LBBM) by 50% compared with control cells (Lavenus et al., 2020). During active LBBM the vimentin is concentrated in the cell body, and the bleb is formed in the vimentin-free area (Adams et al., 2021). This means that loss of vimentin or its redistribution into the perinuclear space under the influence of any antitumor agents can cause the transition to amoeboid movement. Molecular mechanisms provoking the transition to amoeboid movement in case of vimentin destruction are not clearly understood. It was shown that, in some cases, disruption of the VIF network leads to an increase in acto-myosin contractility. Thus, both knockout and siRNA-mediated depletion of vimentin was shown to increase the activity of RhoA-specific Rho GTPase exchange factor GEF-H1 and subsequent led to RhoA activation and phosphorylation of the myosin light chain, resulting in increased actomyosin contractility (Jiu et al., 2017). However, another study showed that in mouse embryonic fibroblasts, with VIF knockout, acto-myosin contractility is significantly reduced (Vahabikashi et al., 2019). It also has been shown that vimentin suppression leads to impairment of FA formation (Tsuruta and Jones 2003; Ivaska et al., 2007; Ostrowska-Podhorodecka et al., 2021; Ostrowska-Podhorodecka and McCulloch, 2021), which in turn can disrupt the integrity and structure of the submembrane actin cortex and cause MAT. In addition, it has recently been shown that VIF and F-actin have extensive structural interactions within the cell cortex and form interpenetrating networks (Wu et al., 2022). Therefore, vimentin deficiency can lead to changes in the properties of the actin cortex, which, as we discussed above, can in turn serve as a trigger for the transition to amoeboid movement. Thus, the search for new antitumor agents whose action is aimed at suppressing migration by disrupting the VIF network seems quite promising, but it should be taken into account that these agents may have side effects such as the emergence of migratory plasticity and provoke switch to amoeboid migration.

## 6 Cytoskeletal proteins as predictive markers of possible transition

Stopping the metastasis is an important task and many works are devoted to attempts to identify special markers of metastasis (Gerashchenko et al., 2019). Most of these markers indicate the presence of an invasive front in the tumor and its ability to migrate, mostly by mesenchymal motility mode. Based on these markers, tumor type is determined and a personalized therapeutic approach to treat tumor treatment could be chosen. However, as we have discussed, the presence of migratory plasticity in tumor cells can seriously compromise treatment.

It is important to predict possible undesirable effects of therapy when developing a treatment strategy for a particular tumor. The manifestation of migratory plasticity and the ability of tumor cells to acquire the ability to migrate or to exchange their movement modes are among these undesirable effects. When selecting treatment scheme, it is important to be able to predict which tumor may exhibit migratory plasticity under the influence of certain therapeutic approaches. Cells migrating by the mesenchymal mechanism express a number of specific proteins, by the presence of which one can say that the given tumor has undergone EMT and its cells have switched to mesenchymal movement. There are almost no such markers for amoeboid movement. And even less is known about markers that could predict the possibility of cells to switch from one type of movement to another. Below we discuss recent data on what tumor characteristics can tell us about its ability to migratory plasticity (Table 2).

**TABLE 2:** Markers to predict tumor migration plasticity.

Markers	Tumor	References
Alteration of morphological features		
Pronounced intratumor morphological heterogeneity, presence of CSC (Cancer Stem Cells)	Breast cancer	Gerashchenko et al. (2019)
	Liver cancer of different morphological subtype (SNU-475 and HepG2 liver cancer cell lines)	Nguyen et al. (2022)
Blebbing of patient tumor cells in *vitro* test	Prostate cancer	Khan et al. (2019)
	Suggested for tumors of different origin	Chikina et al. (2019a)
Proteins associated with the ability to migratory plasticity		
filamin A	Triple negative breast cancer	Zhou et al. (2022)
	Melanoma	Zhang et al. (2014)
	Breast cancer	Jiang et al. (2013)
	high-grade astrocytoma or metastatic breast cancer	Alper et al. (2009)
fascin 1 (actin-bindling protein)	hypopharyngeal squamous cell carcinoma (HSCC)	Bu et al. (2019)
	oral squamous cell carcinoma (OSCC)	Alam et al. (2012)
	Tumors of different origin	Reviewed in Liu et al. (2021)
Podoplanin (PDPN) (increase of blood soluble fraction)	Tumors of different origin	Reviewed in Quintanilla et al. (2019)
	metastatic human melanoma cell line	de Winde et al. (2021)
	melanoma	Zhao et al. (2018)
α-actinin-1 (ACTN1)	basal-like breast cancer	Kovac et al. (2018)
α-actinin-4 (ACTN4)	Advance stages of ovarian cancer	Yamamoto et al. (2009)
	Tumors of different origin	Reviewed in Tentler et al. (2019)

Heterogeneity of tumor cell composition may be a common sign of high tumor aggressiveness and particularly the ability to dissemination (Gerashchenko et al., 2019; Nguyen et al., 2022). In particular, the presence of tumor stem cells can be a sign of possible metastatic activity and propensity of cells for migratory plasticity. The relationship between stemness and the ability of cells to both EMT and further MAT has been described in many works (Taddei et al., 2014; Jolly et al., 2015; Chikina et al., 2019a; Rodriguez-Hernandez et al., 2020).

Above, we described which cytoskeletal proteins have a significant influence in determination of cell migration modes. To use these proteins as markers to assess the probability of migratory plasticity in tumor, we need to be able to assess changes in their expression level using a minimally invasive assay.

For example, the increased expression of Fascin1 or filamin A, which may indicate the potential for migratory plasticity of tumor cells, could be detected in blood (Adams et al., 2021). Fascin is considered in many works as a potential prognostic biomarker for breast, colorectal, esophageal cancers and head and neck squamous cell carcinomas, in hypopharyngeal squamous cell carcinoma (HSCC) (Bu et al., 2019). It was assumed as significant independent negative prognostic factor for survival after multivariate analysis include cancers of the liver, ovary, lung, pancreas, colorectal, head and neck squamous cell carcinoma, and brain (Machesky and Li, 2010). It can also be suggested as a prognostic factor for determining the potential migratory plasticity of tumors.

Another protein, the presence of which may indicate the ability of cells to migratory plasticity, is podoplanin. Podoplanin (PDPN, other names are PA2.26, gp38, D2-40, and Aggrus), is a small very conserved across vertebrates transmembrane mucin-like glycoprotein. Podoplanin expression is associated with myofibroblast phenotypes and upregulated during inflammation and cancer (Quintanilla et al., 2019). Podoplanin drives actomyosin contractility through the activation of RhoA/C GTPases in lymphoid fibroblasts (Martyn-Villar et al., 2006; Acton et al., 2014) and its high expression correlates with poor prognosis and higher incidence of metastatic disease (Quintanilla et al., 2019).

In recent studies performed in the V. Sanz-Moreno’s laboratory, it was shown that high PDPN mRNA expression determine the high metastatic status of the metastatic human melanoma cell line WM938B compare to its non-metastatic counterpart WM938A (de Winde et al., 2021). Its expression drives contractile amoeboid morphology in melanoma, thus triggering invasion and promoting metastasis (de Winde et al., 2021). The cells with increased expression of podoplanin demonstrated active invasion by amoeboid type migration both *in vivo* and *in vitro*. High podoplanin expression has been observed at the invasive front of tumors of different cancer types (Quintanilla et al., 2019), where individual invading amoeboid cells are also observed (Cantelli et al., 2015; Pandya et al., 2017; Georgouli et al., 2019). The levels of soluble podoplanin in plasma were found to increase in patients with different kind of cancers compared to normal individuals, as well as in patients with metastasis with respect to patients with non-metastatic tumors (Zhao et al., 2018).

## 7 Discussion

The importance of developing new anti-tumor approaches aimed at halting metastasis is becoming increasingly evident. The relevance of this area is confirmed by the fact that the FDA proposed to introduce the metastasis free survival of patients (MFS) as a valid endpoint in clinical trials to evaluate the success of treatment of solid tumors (Beaver et al., 2018). New data obtained in recent years in the study of migratory plasticity have shown the need to take this phenomenon into account in the development of therapeutic approaches and the search for new drugs for cancer treatment (Solomon et al., 2021). The development of new drugs based on the effect on cytoskeletal alterations underlying cell migration is an interesting but very challenging task, a detailed review of recent studies is (Ruggiero and Lalli, 2021). And one of the main problems is the manifestation of migratory plasticity which allows cancer cells to escape treatment and to lead to increased metastasis.

Despite the large number and variety of triggers that can cause reciprocal transitions between different modes of movement, the main factors regulating the transition to amoeboid movement are 1) the growth of intracellular hydrostatic pressure under the effect of increasing contractility, regulated by the Rho-ROCK-MLCK signaling pathway (Lawson and Ridley, 2018; Guan et al., 2020; Crosas-Molist et al., 2022) or due to the cell getting into specific confined conditions during migration and 2) changes in the integrity of the actin cortex cytoskeleton or its interaction with the cell membrane. Confinement conditions can be modeled in *vitro* experiments (Liu et al., 2015; Ibo et al., 2016), but also can occur during long way cell migration either at places of high contractility within the gastrulating embryo (Ruprecht et al., 2015), or during cancer cells dissemination from the primary tumor to secondary lessions (Paul et al., 2017; Yamada and Sixt, 2019). Recently, several studies have shown that the cell nucleus may be an essential mechanosensor determining the mode of migration. According to these findings, the progressive nuclear deformation, induced by cell confinement, triggers intracellular events that promote cell contractility and determine the transition to fast amoeboid migration (Lomakin et al., 2020; Venturini et al., 2020; Lavenus et al., 2022).

Since we now know a lot about the basic mechanisms that regulate switching between different modes of motility, it seems rational, to use either agents that inhibit all modes of migration or simultaneously several agents that would prevent a possible transition in cases where we can suspect that cells of a given tumor may exhibit migratory plasticity. For example, it has been proposed that simultaneous inhibition of ECM proteolysis and Rho-ROCK-MLCK2 pathway could be an effective approach to stop the dissemination of cancer cells (Sahai and Marshall, 2003). Rho inhibitors represent a promising possibility for stopping tumor cell migration and metastasis (Clayton and Ridley, 2020). Cell movement using both mesenchymal and amoeboid mechanisms requires the coordinated work of protrusions and contraction, that’s why disruption of the contractile component can stop movement by any motility mode (Sadok et al., 2015; Rodriguez-Hernandez et al., 2016). Such compaunds as ATP-competitive AKT kinase inhibitors, AT13148 and CCT129254 which also inhibit the Rho-kinases ROCK 1 and ROCK 2 were suggested to stop tumor cells dissemination. It was shown that AT13148 potently inhibit ROCK activity in melanoma, neuroblastoma and pancreatic cancer cells in culture and *in vivo* (Rodriguez-Hernandez et al., 2016; Dyberg et al., 2017; Rath et al., 2018). These compounds have shown encouraging results in the preclinical trials. But after clinical trials the use of AT13148 has not been recommended because of its numerous side effects and negligible therapeutic effect (McLeod et al., 2020). It should be taken into account that inhibition of enhanced acto-myosin contractility, aimed at stopping the amoeboid movement, can lead to an increase in the migration abilities of cells due to the activation of mesenchymal movement. For example it was shown that inhibitor of ROCK Y-27632 increased the invasiveness of human glioma U87 and U251 cells (Salhia et al., 2005) and also enhanced the invasion of human gastric carcinoma OCUM-2MD3 cells (Matsuoka et al., 2011). Targeting the regulatory pathways has its disadvantages because the main regulatory pathways are involved in many functions and their suppression provides a field for the development of numerous side effects. In addition, because due to the complexity of regulation, bypass pathways and thus resistance to used drug can develop. The development of agents aimed specifically at stopping cell migration is actively discussed. Thus, a new term migrastatic is proposed for drugs directed against all types of migration, the action of which is based on inhibition of acto-myosin contractility and actin polymerization as downstreams effector mechanisms (Solomon et al., 2021) that are common and essential for the motility of all migrating cancer cells (Gandalovičová et al., 2017). The using of cytoskeletal proteins that regulate migratory plasticity as targets of therapeutic therapy seems to be a promising direction for the development of new approaches in cancer treatment.
